# Is the creation of artificial life morally significant?

**DOI:** 10.1016/j.shpsc.2013.05.016

**Published:** 2013-12

**Authors:** Thomas Douglas, Russell Powell, Julian Savulescu

**Affiliations:** a Oxford Uehiro Centre for Practical Ethics, Suite 8, Littlegate House, St. Ebbes Street, Oxford OX1-1PT, United Kingdom; b Department of Philosophy, Boston University, 745 Commonwealth Avenue, Boston, MA 02215, USA

**Keywords:** Artificial life, Function, Genetic engineering, Moral status, Reductionism, Synthetic

## Abstract

In 2010, the Venter lab announced that it had created the first bacterium with an entirely synthetic genome. This was reported to be the first instance of ‘artificial life,’ and in the ethical and policy discussions that followed it was widely assumed that the creation of artificial life is in itself morally significant. We cast doubt on this assumption. First we offer an account of the creation of artificial life that distinguishes this from the derivation of organisms from existing life and clarify what we mean in asking whether the creation of artificial life has moral significance. We then articulate and evaluate three attempts to establish that the creation of artificial life is morally significant. These appeal to (1) the claim that the creation of artificial life involves playing God, as expressed in three distinct formulations; (2) the claim that the creation of artificial life will encourage reductionist attitudes toward the living world that undermine the special moral value accorded to life; and (3) the worry that artificial organisms will have an uncertain functional status and consequently an uncertain moral status. We argue that all three attempts to ground the moral significance of the creation of artificial life fail, because none of them establishes that the creation of artificial life is morally problematic in a way that the derivation of organisms from existing life forms is not. We conclude that the decisive moral consideration is not how life is created but what non-genealogical properties it possesses.

## Highlights

• Artificial organisms need not pose greater risks than derived organisms.
• The creation of artificial life would not support any form of biological reductionism.
• Artificial life forms might have uncertain functional status.
• But this is irrelevant to their moral status.
• The genealogy of an organism makes no difference to its moral status.

In 2010, staff at the J. Craig Venter Institute (JCVI) reported the creation of the first bacterium with an entirely synthetic genome. A variant on the *Mycoplasma mycoides* genome was stitched together from simple chemical building blocks and then inserted into a bacterium from a different *Mycoplasma* species whose genetic contents had been removed. The result was a novel bacterium that was capable of reproducing and performing other normal bacterial functions (Gibson et al., 2010).

The JCVI’s creation was widely reported as the first example of artificial life. It is doubtful whether it was aptly characterised as such, given that only the genome and none of the cytoplasmic structures were synthesised by scientists (Bedau et al., 2010). However, in this paper we take no committed stance on whether the JCVI’s bacterium constituted artificial life. Instead, we consider whether it would have mattered, morally, if it did. Even if JCVI scientists did *not* create artificial life, this is an important question to ask since future scientists may well do so.

In the controversy that surrounded the JCVI’s creation, which prompted a meeting of the U.S. Presidential Commission for the Study of Bioethical Issues and an ensuing ethical report (2010), it was widely assumed that the creation of artificial life *is itself* morally significant. In this article we aim to cast doubt on this assumption. First we offer an account of what it is to create artificial life and clarify what we mean in asking whether the creation of artificial life has moral significance. We then articulate and evaluate three attempts to establish that the creation of artificial life is morally significant: one based on the concern that the creation of artificial life involves playing God, one claiming that it will encourage reductionist attitudes toward life, and one which argues that artificial organisms will have uncertain moral status. We show that all three attempts fail.

## 1 Definitions

We shall first lay out some key definitions.

### 1.1 Creating artificial life

The creation of artificial life would have to consist, we assume, in the creation of an artificial *living entity*, henceforth an ‘artificial organism’.^1^ We will take an organism to be artificial just in case *either* (1) all core elements of that organism were initially constructed from chemically simple, non-living components to the specification of a person or other natural rational being,^2^ *or* (2) it descended from an organism (or pair of organisms in the case of sexual creatures) that was constructed in this way.

There are several points to note about this definition. First, it does not require that to qualify as artificial, an organism must be novel in kind—by which we mean, substantially different in kind (genetically or phenotypically) to any previously or currently existing organism. Had the JCVI’s creation been genetically and phenotypically identical to the wild type *Mycoplasma mycoides*, this would not have affected its artificiality. Second, whether the JCVI bacterium qualifies as artificial, on this definition, will depend on whether the nuclear genome is the only ‘core element’ of a bacterium—an assumption that many biologists would reject, given the crucial developmental and homeostatic role played by various cellular structures in the cytoplasm and membrane. Third, the qualified phrase ‘*initially* constructed’ is necessary because, once organisms are ‘up and running’, they will frequently be capable of maintaining themselves though exerting a causal influence on their internal and external environment (Saborido, Mossio, & Moreno, 2011). Whereas the parts of rationally designed machines usually wear out with use, organisms will typically renew their parts until death. We take it that if an animal were artificial when first created, it would remain artificial at the end of its life. Fourth, we employ the disjunction ‘or it descended from … ’ to accommodate our view that a reproductive lineage descending from an artificial organism or pair of organisms remains artificial in perpetuity since it reflects a continuous causal process that originates in a single artificial creation event (more on this below). And finally, fifth, we note that the above definition of artificiality does not cover domesticated plants and animals that result from selective breeding, or even genetically modified organisms (GMOs), since these are not constructed from chemically simple, non-living materials.

### 1.2 Moral significance

Since we wish to assess the claim that the creation of artificial life is morally significant, it is necessary to say something about how we understand moral significance. There are various ways in which this concept might be understood, but we wish to capture how it has been invoked in discussion of the creation of artificial life. There, the thought has typically been that the creation of artificial life is morally significant in a fundamentally *negative* way. We will take it to have such significance just in case (a) there are moral reasons not to create artificial organisms, or factors that weaken our moral reasons *to* create them, and (b) these are specific to the creation of artificial organisms.^3^ The second requirement, holding that the factors which bear negatively on the creation of artificial organisms be specific to this practice, requires some further elucidation. It implies that there are some contrasting practices to which these factors do not apply. But what are those contrasting practices?

The creation of artificial organisms is most naturally contrasted with the much more familiar practices whereby one organism is derived from one or more others. People derive new organisms from pre-existing ones, for example, by engaging in sexual relations with one another, undergoing or providing fertility treatments, harvesting and planting seeds. In some cases, as in most instances of normal human reproduction, the resulting organisms satisfy neither of the conditions for artificiality that we introduced above—they are neither created from chemically simple components, nor created to the specifications of a rational agent. In other cases, the new organisms that we derive from existing ones *are* created to the specification of one or more rational agents who envision a set of desirable organismic properties and manipulate living processes to achieve (or approximate) that end. This is true of domesticated plants and animals developed through selective breeding programs. It is also true of GMOs, as well as organisms that are generated via ‘directed evolution’ in the laboratory, where desirable properties are intentionally selected, rather than engineered in accordance with rational engineering principles (see O’Malley, 2011).

Since the production of new organisms to the specifications of rational agents is already widespread (and indeed has been since the Agricultural Revolution), it would be surprising if those alarmed by the prospect of creating artificial life were alarmed by the created-to-specification aspect of artificial organisms. If they were, it would be difficult to explain why the JCVI’s creation was singled out for attention. It seems more plausible to assume, then, that what alarms some has specifically to do with the fact that artificial life forms would be produced to specification *from chemically simple, non-living components*.

This interpretation is supported by the emphasis that some authors have placed on the claim that creating artificial life is ontologically more radical than the mere derivation or manipulation of living things. Boldt and Müller (2008) put the point this way:

The shift from genetic engineering’s ‘manipulatio’ to synthetic biology’s ‘creatio’ is a shift with considerable ethical significance … . In synthetic biology, the aim is not to amend an organism with a certain quantity of altered characteristics (that is, to manipulate); instead, it is to equip a completely unqualified organism with a new quality of being (that is, to create a new form of life).

The ethically relevant contrast for artificial life, therefore, appears to be the derivation of organisms from other life forms, irrespective of the mode of derivation and regardless of whether it is carried out to human specification.

We can now refine our conditions for moral significance as follows. The creation of artificial life has moral significance just in case (a) there are reasons not to create artificial organisms, or factors which weaken our reasons *to* create them, and (b) these reasons or factors would not apply—or would not apply with equal force—to the derivation of similar life forms from previously existing life forms. ‘Similar organisms’ should be understood as referring to organisms possessing similar non-genealogical properties. ‘Derivation’ should be understood as describing the generation of a new organism through the modification of a continuous causal process (for example, an unbroken chain of reproduction, cellular mitosis, epigenesis, *et cetera*) that extends over space and time. Modifications transmitted via a continuous casual process include, for example, intentional genetic alterations and random sexual recombination events.

The creation of a minimal bacterium by the JCVI stands in stark contrast to the derivation of life. Although the JCVI’s synthetic genome contained only minor deviations from the naturally occurring *M. mycoides* genome, and was in some *informational* sense ‘derived’ from the latter, the synthetic genome was not part of a continuous causal process of genetic transmission linking the wild type organism with the derived organism. Rather, it was built *de novo* from basic biological components, and then inserted into the cytoplasm of an entirely different species of bacteria whose DNA had been removed.

It is this element of ‘creatio’—the generation of novel organisms *de novo*—that many authors find ethically troublesome in relation to synthetic biology, and which could potentially form the basis of an argument that the creation of artificial life is morally significant. In what follows we develop and assess three prominent attempts to ground the moral significance of creating artificial life. We find each of them wanting.

## 2 Attempt one: playing God

An initial attempt to establish the moral significance of creating artificial life is suggested by the reception, in the popular press, of the JCVI’s creation. A common refrain in this literature is that the Institute’s staff members were ‘playing God’ (see, e.g., Alleyne, 2010; McCrae, 2010; Savulescu, 2010). Concerns about playing God have been raised in relation to many different areas of the life sciences, medicine and environmental policy, and though these concerns have not been developed systematically in the philosophical literature, there has been some philosophical treatment of them (see, for example, Coady, 2009; van den Belt, 2009). On the basis of this treatment, it is possible to delineate at least three different variants of the concern about playing God. All of these could, with some initial plausibility, be raised in relation to the creation of artificial life.

An overtly religious variant of the playing God concern maintains that the putatively worrisome practice involves literally usurping the proper role of some higher being or god. This worry was raised in the context of the recombinant DNA project that emerged in the 1970s (Goodfield, 1977) and it has been raised with some regularity in response to more recent attempts to modify organisms through genetic engineering (Evans, 2002). This version of the playing God concern maintains that humans should not intervene in certain ‘forbidden’ realms of the natural world regardless of what the likely consequences of such interventions will be.

A second, fundamentally secular version of the playing God concern maintains that agents who engage in the practice in question thereby express objectionably grandiose or hubristic attitudes. This worry has been raised, for example, in relation to the use of biomedical technologies to enhance one’s normal capacities or to produce ‘designer children’ through the genetic selection or modification of human embryos (Kass, 2003; Sandel, 2007). It is argued that these practices express arrogance and a drive to mastery when the appropriate attitudes to take towards one’s capacities and one’s children are attitudes of acceptance and gratitude (what Sandel calls ‘giftedness’). Since this version of the playing God concern focuses on the attitudes of the agent said to play God and not on the consequences of his/her doing so, it sidesteps empirical debates over the likely psychological, social, and environmental consequences of the practice in question (Buchanan, 2011).

A third, also secular version of the playing God concern is explicitly outcome-based, maintaining that the practice or intervention in question involves overstepping the limits of human knowledge, thus unwarrantedly risking unintended negative consequences. Complex living systems are taken to exist in an optimal but delicate equilibrium which human intervention is likely to disrupt (Powell, 2010).

We shall set aside the theological variant of the playing God concern and focus instead on the two secular variants. It is plausible that the creation of artificial life could, in *some* cases, raise both of these concerns. It could express grandiose or hubristic attitudes, and it could unwarrantedly risk negative consequences. It is highly doubtful, though, whether the creation of artificial life would *always* (or even often) raise these concerns. For example, it is implausible that any scientist who creates artificial life in order to produce an effective pharmaceutical or a targeted medical treatment *must* harbour grandiose attitudes or pose undue risks. What is more important in the present context, however, is that it is not clear that these two secular concerns about playing God apply more powerfully to the creation of artificial life than they do to the derivation of similar life.

Consider first the risk-based variant of the playing God concern. The seriousness of this concern, when raised in relation to the production of new organisms, will depend on the level of risk posed by the organism produced.^4^ But the risk that an organism poses depends not on its etiology, but on its causal properties—that is, how it interacts with other organisms and the environment. Organisms derived from a dangerous pathogen like *Bacillus anthracis* (which causes anthrax) are obviously far more dangerous than artificial versions of a benign microbe involved in the fermentation of dairy products. There is no reason to suppose that two organisms with the same causal properties will pose different risks merely because one was artificially created while the other was not.

At this point, it might be argued that we have understood the risk-based variant of the playing God concern too narrowly. The worry is not that particular instances of creating artificial life will invariably pose greater risks than the derivation of similar life. Rather, the worry is that creating artificial life is a type of practice that would be associated with greater *average* or *maximal* risks than the derivation of similar life. Because *creatio* involves greater degrees of freedom than *manipulatio*, artificial organisms could, and perhaps typically would, depart more radically, genetically and phenotypically speaking, from the range of existing organisms than would derived organisms. And perhaps this more radical departure from existing variation would be associated with higher average or maximal levels of risk to human health or the environment.

One line of reasoning for this last view holds that endemic species, communities and ecosystems are at high risk of being ravaged by invaders, since they are not adapted to defend or compete against these ‘alien’ intruders. The more alien the invaders are, so the logic goes, the less likely endemic species or communities will, as ‘naïve’ prey or competitors, be able to resist the novel predation or competition pressures introduced by the invaders. Because artificial organisms would typically be more alien than derived ones, their creation would typically pose greater risks.

An opposing and arguably more plausible view is that the risk of antagonistic interactions between introduced and endemic organisms is actually less the more alien the introduced organism is. This is because strategic co-evolution, such as that characterizing predator–prey (and host–parasite) dynamics, typically requires specialized adaptations that can only evolve through prolonged evolutionary contact between lineages. One reasonable tentative conclusion, therefore, is that artificial organisms that differ radically from existing ones would, generally speaking, pose lesser risks to endemic fauna than organisms that differ less radically or even only slightly from their natural counterparts, since radically different artificial organisms would be unlikely to compete over niches with naturally existing organisms.^5^ These evolutionary considerations support scepticism regarding the claim that artificial organisms would typically pose levels of risk greater than those posed by derived organisms.

There are also reasons to be sceptical about the suggestion that artificial organisms would pose greater *maximal* risks than derived organisms. It is possible that artificial organisms could pose extreme risks that involve an existential threat to humanity. But the same is true of derived organisms. Indeed, currently existing organisms, such as smallpox or Ebola, already pose extreme, possibly existential risks, and such organisms could be genetically modified so as to make them even more lethal.

More importantly, even if the creation of artificial life would pose greater average or maximal risks than the derivation of life, it is not clear that this would have any moral significance. This is because an interpretation of the risk-based playing God concern which focuses on the average or maximal level of risk that a type of activity poses lacks much of the intuitive appeal of the original, narrower interpretation which focuses on the *actual* level of risk posed by a particular activity; it ends up using a sledgehammer where a scalpel will do. If a particular instance of creating artificial life were associated with risks of negative consequences, it could be morally problematic. However, it is difficult to see why the creation of artificial life should be thought morally problematic in cases where it is *not associated with such risks* merely because *other* instances of creating artificial life commonly *are* risky. The plausibility of the risk-based variant of the playing God concern derives from the thought that there are moral reasons not to risk negative consequences, not from the thought that there are moral reasons not to engage in practices that are typically risky.

One might claim that we are not in an epistemic position to divine risk in individual cases, and thus we must rely on an average assessment of risk. But this is certainly not the case in the context of derived organisms (compare, e.g., the derivation of cattle from the derivation of cholera), and thus it is not clear why it should be the case for artificial organisms.

Alternatively, one might claim that, even if we can distinguish lower- and higher-risk artificial organisms, we could not create the lower-risk variants without also facilitating the creation of higher-risk variants. Thus, we should assess a particular instance of creating artificial life in part by assessing the average or maximal risks associated with the other instances of creating artificial life that it might facilitate. But again, we can frequently *derive* low-risk organisms without contributing to the derivation of high-risk ones, and it is unclear why the situation should be any different for the creation of artificial organisms.

In short, the risk-based variant of the playing God concern is most forceful when understood narrowly, but understood thus, it is highly doubtful that it applies specifically to the creation of artificial life—and hence it is highly doubtful that it can ground the claim that the creation of artificial life is morally significant. What matters is the nature of the life in question and its causal powers; not how it was derived.

Similar thoughts apply to the expressivist variant of the playing God objection. Understood narrowly, this variant would apply more powerfully to the creation of artificial organisms than to the derivation of similar organisms only if the former would express more objectionable attitudes than the latter. But what attitudes the production of an organism expresses plausibly depends on what attitudes the creator of that organism actually has, not on the nature of the means via which the organism was created, and there seems no reason to suppose that a synthetic biologist who creates an artificial organisms must hold more objectionable attitudes than a molecular biologist who modifies an existing organism. Both scientists could (though need not) be driven by arrogance or other grandiose motives, and both scientists could (though need not) be motivated by a desire to master nature. Perhaps it could be argued that the very same motives and attitudes that would be unobjectionable in a scientist seeking to modify or manipulate existing organisms would be objectionable in a scientist seeking to create artificial life, but it is unclear what could justify drawing such a distinction.

By contrast, on a broader interpretation of the expressivist concern, the concern applies more powerfully to the creation of artificial life than to the derivation of life provided the former is *typically* associated with more objectionable attitudes than the latter. Moreover, it is not clear why we should accept broader interpretation of the concern. If a particular instance of creating artificial life were associated with objectionable attitudes, it is at least somewhat plausible to say that it would, as a result, be morally problematic. However, it is difficult to see why the creation of artificial life should be thought morally problematic when not associated with objectionable attitudes merely because *other* instances of creating artificial life are associated with such attitudes.

Like the risk-based variant of the playing God concern, the expressivist variant is most forceful when understood narrowly, but understood thus, it is highly doubtful that it applies specifically to the creation of artificial life—and hence it is highly doubtful that it can ground the claim that the creation of artificial life is morally significant.

## 3 Attempt two: encouraging reductionism

In an early paper on the ethics of synthetic biology, (Mildred Cho and collaborators, 1999) set forward another possible ground for the moral significance of creating artificial life. They suggest that the creation of artificial life could lead to the widespread acceptance of a reductionism about life according to which life is “nothing more” than a set of biochemical components, or, more restrictively, a set of genes. They worry that this, in turn, might undermine “the special status of living things and the value that we ascribe to life”. The claim, it seems, is that the knowledge generated by and practices associated with synthetic biology will cause people to no longer regard the distinction between living and non-living things as important. But—so the argument goes—this distinction *is* important, for it marks a difference in value: Living things possess, in virtue of being alive, some value that non-living things lack. We have moral reasons not to bring it about that people assign no significance, or less significance than is due, to this moral distinction. Thus, we have moral reasons not to create artificial life.

Is this concern peculiar to the creation of artificial life? Cho and collaborators do not go on to contrast the creation of artificial life with the derivation of similar life, but one can imagine how such a contrast might be drawn. On their view, the creation of artificial life makes it difficult to sustain the belief that there is something more to life than a certain kind of arrangement and interaction of chemical building blocks: the thought is presumably that it will be difficult to explain how this ‘something more’ could reliably be produced merely by arranging chemical components. By contrast, there is arguably no comparable problem posed by the demonstration that one living thing can be derived from another, for in that case, one may suppose that the ‘something more’ property is transferred from the ancestral organism to its descendant organism.

Reductionism about life can be understood as an ontological thesis or an epistemic claim about explanation or inter-theoretic relations. Virtually all biologists and philosophers of biology are physicalists—they maintain that all things in the universe are either physical or supervene on (roughly: are determined by) the physical. However, very few philosophers of biology endorse *any* version of the reductionist thesis. They almost universally reject the notion that general biological theories (such as the theory of natural selection or Mendelian genetics) can be reduced to physico-chemical theories, even in principle. They are equally sceptical that biological explanations, which refer to biological kinds such as species, populations, organisms, traits, cells, genes *et cetera*, can be successfully translated into explanations that cite only molecular biological kinds. The failure of theoretical and explanatory reductionism in biology is not a symptom of human epistemic limitations, but rather a reflection of the genuinely hierarchical structure of the biological world (Kitcher, 1984)—in other words, these forms of reductionism fail because ontological reductionism is false.

Whether the simultaneous commitment to physicalism and anti-reductionism on all fronts poses a conflict that biologists and philosophers of science need to reconcile is beyond the scope of this article (for a discussion, see Rosenberg & Kaplan, 2005). What matters for our purposes is this: even if creating artificial life demonstrates that the physico-chemical facts fix the biological facts, all this shows is that biological properties supervene on physical properties—a version of the physicalism thesis that essentially every philosopher of science and biologist already accepts.^6^ Only a vitalist would dispute this. The creation of artificial life may thus be viewed as contradicting vitalism, but vitalism was discredited as an unscientific and empirically unsupported hypothesis long before synthetic biology came along.

One might argue that the creation of artificial life would support *methodological* reductionism—a research strategy holding that the most effective way to investigate the properties of complex systems is to decompose them into their lowest level parts. Synthetic biology has often taken this reductionist approach by emphasizing rational design principles that make use of standardized parts with well-understood properties, such as biobricks™. But synthetic biologists also frequently rely on directed evolution to tap into subtle causal interactions that are invisible to genetic engineers and recalcitrant to standard engineering manipulation—suggesting that the reductionist approach to building organisms to specification may be of limited efficacy (for a discussion see Lewens, this issue), in part due the emergent properties of complex systems (Bedau, 2008). If synthetic biology is supposed to support the methodological thesis of reductionism by showing that the properties of the whole can be predicted and controlled by understanding the properties of the parts, it has done so with limited success. Even if it were successful, however, it is not clear why a reductionist scientific methodology should have any implications whatsoever for ethics.

We have been assuming that Cho *et al.* are concerned about the possible reduction of biological properties to physico–chemical properties. However it may be that they have a different kind of reductionism in mind. For example, perhaps they are concerned rather about the ontological reduction of *organismic* properties to *genetic* properties. As we noted, virtually no biologist would accept a metaphysical theory that relegates organisms, traits, and cells to the illusory—and what’s more, there is no reason to immunize genes from this relegation, given that the ontological status of the gene is heavily contested (see Griffiths & Stotz, 2006). Neither would anyone claim that organismic properties are identical to genetic properties. Genes do not determine organismic properties in the way that sub-atomic elements determine the physical properties of atomic elements. And unlike neuronal states which may be synchronic with mental states, genetic states are well removed in space and time from, and thus cannot be identified with, their ultimate effects on the phenotype, which are mediated by a patchwork of intervening causes.

Alternatively, perhaps Cho and collaborators are concerned that the creation of artificial life would support a kind of *explanatory* genetic reductionism according to which the phenotypic properties of an organism are to be causally explained wholly or primarily by reference to the organism’s genes. At first glance, the success of JCVI’s synthetic genome does seems to imply that genes are a controlling causal influence on the development of organismic properties, as compared say to the cytoplasmic structures of the host bacteria which was outfitted with the synthetic genome. The mere fact that genes are acting as causal difference-makers in *this* case, though, need not generalize to other cases, such as to the traits of complex animals, which rely more heavily on environmental interaction for their development. More importantly, however, the genetic manipulation of existing genomes has demonstrated the causal influence of genes with equal rigour: consider, for example, genetic interventions that have been shown to induce the development of ectopic organs in fruit flies, such as a compound eye where a wing ordinarily would be. For these reasons, it is not clear that synthetic biology offers a uniquely powerful demonstration of genetic reductionism in any of its variations.

Finally, Cho and collaborators might be interpreted as maintaining not that the creation of artificial life *actually* supports any of the abovementioned reductionist theses, but that it would be *perceived* to support them. For instance, suppose it were the case that people who believed that the property of being alive supervened on biochemical properties tended also to believe that life is no more than a collection of biochemical components, even though this is false. If so, then to the extent that artificial life demonstrates supervenience relations, it might engender the perception that ontological reductionism is true.

There are, however, at least two serious problems with this line of reasoning. First, in the absence of empirical evidence that people are prone to draw mistaken inferences of this sort, it would seem uncharitable to assume that they will. Second, even if the creation of artificial life were to lead to widespread acceptance of some form of reductionism, it is not immediately clear why this would undermine “the special status of living things and the value that we ascribe to life”, as Cho and collaborators fear (Douglas & Savulescu, 2010). Humans and other beings with moral status are typically ascribed their special value on grounds other than that they are alive. It is very doubtful, for example, that bacteria and protozoa have any moral status (or even value) merely in virtue of their being alive; most of us would judge that there is nothing wrong with killing the numerous bacteria lining our skin and intestine or the weeds in our garden, let alone the flies buzzing against our windows. Rather, moral status is typically attributed to beings in virtue of the mental capacities they or normal members of their species possess. Typically these are capacities for consciousness, experiencing pleasure and pain, self-consciousness or rationality. Since it is a psychological life, rather than biological life, that is taken to confer moral status, it is not immediately clear why acceptance of reductionism about life should have any impact on our inclination to ascribe such status.

Moreover, even if the view that merely being alive confers no moral status were false, it is not clear that we would have much to fear from the creation of life. If this is a false view, it is a false view that virtually everyone already accepts, at least implicitly, and those who reject it do so on the basis of sophisticated philosophical theories that are unlikely to be affected by the creation of artificial life. Thus, it is unlikely that the creation of artificial life would make any difference to the number of people who hold the view.

Perhaps what Cho and collaborators are concerned about is that acceptance of reductionism would engender a frivolous attitude towards life: people might start creating new life forms willy–nilly, in much the same way that children might create different coloured compounds with elementary chemistry sets, which is inconsistent with treating life as something that is ‘sacred’ or worthy of respect. Perhaps this would be problematic even if the life forms created lacked moral status. We have already noted, however, that merely being alive is not ordinarily thought to demand respect; moreover, life can easily be created now using sexual reproduction and it can be radically modified using breeding or genetic engineering. It is thus hard to see how synthetic biology raises new concerns of this sort.

In sum, it is not clear that the creation of artificial life would encourage acceptance of any version of the reductionist thesis. And even if it did, it is not clear that this would lead to the acceptance of any new and incorrect views regarding the value of life.

## 4 Attempt three: an argument from evolutionary teleology

A third attempt to establish the moral significance of creating artificial life draws on the thought, again widely mentioned in discussions of the JCVI creation, that artificial organisms would not fit clearly into our organism–artefact dichotomy, and would thus have or be perceived to have an uncertain ontological status.^7^

The uncertain ontological status of artificial life might create puzzles for biologists and metaphysicians, but it is not immediately clear why this would bear negatively on the morality of creating such life forms. Boldt and Müller (2008, p. 188) suggest that it might be problematic for reasons which parallel those offered by Cho and collaborators. They contend that metaphors often deployed in synthetic biology, such as those comparing DNA segments to “Lego bricks” and artificial life to “living machines”, serve to “identif[y] organisms with artefacts, an identification that, given the connection between ‘life’ and ‘value’, may in the (very) long run lead to a weakening of society’s respect for higher forms of life that are usually regarded as worthy of protection”. However, as we argued in the last section, higher forms of life are not normally ascribed moral status in virtue of their being alive, but in virtue of their possessing certain mental capacities, and it is not clear why undermining the distinction between organisms and artefacts would have any influence on the significance we assign to mental properties. Thus, in this section, we consider whether there might be an alternative way to marshal worries about the uncertain status of artificial organisms into an argument for the moral significance of creating artificial life.

In order to do so, we will understand the worry about the uncertain ontological status of artificial organisms as a worry about uncertain *functional* status. For as we will see, uncertain functional status can lead to uncertain moral status, insofar as moral status is tied to interests, and interests are tied to functions. Many biologists and philosophers of biology would accept that ordinary organisms have a functional status: they can aptly be described as functioning well, or functioning poorly. According to the dominant ‘etiological’ account of function, the function of a trait is the effect for which that trait was selected—that is, the effect that causally explains the proliferation and maintenance of that trait in a population lineage via mechanisms of natural selection (Neander, 1991). We call this account of function *Evolutionary Teleology*. Notwithstanding persuasive arguments for functional pluralism (Amundson & Lauder, 1994), *Evolutionary Teleology* remains the prevailing approach to function in biology.^8^

If one believes that the functional status of ordinary living things depends on their evolutionary etiology in the way specified by *Evolutionary Teleology*, then a puzzle arises concerning what would determine the functional status of artificial organisms, since these do not evolve via a process of natural selection but rather are created *de novo* to the specifications of rational agents.^9^ One possibility is that the functional status of an artificial organism might be determined in the same way as the functional status of non-living artefacts. A non-living artefact is, plausibly, well-functioning to the extent that it realises or acts in accordance with functions that are conferred on it by the rational agents that created or use it. For example, the function of a calculator is to accurately perform mathematical calculations because it was created, and is used, with this goal in mind.

However it seems doubtful whether the functional status of an artificial organism would (or would always) be determined in this way. Suppose that it were possible to create an artificial entity with all of the characteristics of a typical chimpanzee embryo and to develop such an embryo into an entity with characteristics of a typical adult chimpanzee. Suppose further that a scientist created such an artificial chimpanzee for the sole purpose of using it in medical experiments.^10^ It is highly doubtful that we would characterize this artificial chimpanzee as well-functioning in virtue of the fact that it was a good experimental subject (for example, it was docile, a good model for human diseases, and so on). It is tempting to think instead that the functional status of this artificial chimpanzee would be determined by the same considerations that would determine the functional status of similar, non-artificial chimpanzees. However, if the functional status of ordinary chimpanzees is determined by backward-looking *Evolutionary Teleology*, which refers to a history of natural selection for phenotypic effects, then it does not appear that the functional status of a non-evolved being, such as our artificial chimpanzee, *could* be determined in precisely the same way.

Various conclusions might be drawn from this problem (see e.g. Neander, 1996). One possible lesson would be that we should reject the extension of *Evolutionary Teleology* to artificial beings. But this would arguably lead us with significant uncertainty regarding the functional status of such beings. We could claim that their functional status can be determined by reference to the closest possible natural, evolved being. However, as artificial organisms become increasingly different from any evolved life form, reference to the functional status of existing organisms would surely become increasingly irrelevant to the functional status of artificial life. Absent some other means of determining the latter’s functional status, there would be uncertainty in this regard. Moreover, even in a case where an artificial organism does closely resemble some natural, evolved being, one might reasonably doubt why the provenance of that natural being should be relevant to the functional status of its artificial equivalent. Even in this case, there might be significant functional uncertainty.

Uncertainty regarding the functional status of artificial moral patients might have moral significance. Suppose we accept the following Aristotelian view, which would command significant philosophical support:

*Prudential Functionalism:* an organism has an interest in functioning well.

That is to say, it is *good for* an organism to function well, or, equivalently, functioning well is prudentially valuable. If *Prudential Functionalism* is correct, then the uncertain functional status of artificial organisms will imply that it is also uncertain what *interests* that organism possesses, since interests are, on this view, tied to functions. Now suppose that we accept a further view, which would also command significant philosophical support:

*Moral Prudentialism:* an organism’s interests give rise to moral protections (i.e., moral reasons to promote, protect or refrain from setting back those interests).

If *Moral Prudentialism* is correct, then it will follow in turn that it is uncertain what moral protections, if any, artificial organisms enjoy, since moral protections are dependent on interests, and the interests of artificial organisms are uncertain due to their uncertain functional status. In this way, functional uncertainty leads to *moral* uncertainty.

This moral uncertainty creates a risk that artificial organisms might be treated in ways that are morally wrong because the scope or strength of the moral protections enjoyed by those organisms is underestimated. This sort of risk is familiar from other domains that have been well studied by medical and animal ethicists. For instance, it is arguably uncertain what moral protections human embryos, foetuses and neonates, severely cognitively impaired adult humans, and many nonhuman animals enjoy, and this uncertainty creates a risk that such beings are treated wrongfully. Some would argue that abortion, non-treatment of comatose patients, and animal farming practices are examples of wrongful treatment that follow, in part, from the underestimation of the strength and breadth of the moral protections enjoyed by these organisms. Take the case of agricultural animals: cows, pigs and chickens were designed through a deliberate process of selective breeding to serve as a human food resource. Perhaps the unusually (and arguably unjustifiably) harsh treatment of agricultural animals is due in part to their having an uncertain functional status—one that is taken to be tied in part to human interests, rather than generated solely by their own evolutionary history. This might also explain the asymmetrical moral attitudes that many people hold toward the human treatment of wild and agricultural animals, respectively.

Of course, it is not always morally wrong, all things considered, to create organisms at risk of wrongful treatment. This can be seen by considering the case of human embryos and foetuses. Though it is arguably uncertain what moral protections these entities enjoy, and though this arguably creates some risk of wrongful treatment, it is surely often permissible to create human embryos or foetuses. Nevertheless, the risk that an entity will be treated in morally wrongful ways may significantly weaken the case for (or strengthen the case against) creating such an entity. Consider that the breeding of farm animals is more defensible when there is a low risk that those animals will be treated wrongfully than when there is a high risk. Similarly, the creation of artificial organisms might be less defensible than the creation of other organisms whose functional status is less uncertain, and which are thus at lower risk of wrongful treatment.

The argument that we have outlined can be summarized as follows: if the functional status of ordinary, evolved organisms is determined by their evolutionary origins in the way specified by *Evolutionary Teleology*, then the functional status of such organisms can be ascertained by determining whether they possesses those traits that were favoured by natural selection in their ancestors. However, the functional status of artificial organisms cannot be ascertained in this way, and this means that their functional status is uncertain. But, since organisms have an interest in functioning well (*Prudential Functionalism*), it follows that it is uncertain what interests artificial organisms have. And, since interests generate moral protections (*Moral Prudentialism*), this translates into uncertainty about what moral protections artificial organisms would enjoy. This in turn creates a risk that artificial organisms would be treated wrongfully—because the strength or breadth of their moral protections would be underestimated. And finally, this risk weakens the moral case for (or strengthens the moral case against) creating these life forms.

The premises of this argument—henceforth ‘the argument from evolutionary teleology’—might seem to be somewhat plausible when assessed in isolation from one another. However, the conjunction of these premises is not plausible. In particular, acceptance of three of them—*Evolutionary Teleology*, *Prudential Functionalism* and *Moral Prudentialism*—generates implausible results. An ordinary bacterium is an evolved organism which, according to *Evolutionary Teleology*, has a functional status. Since an ordinary bacterium has a functional status, it also, pursuant to *Prudential Functionalism*, has interests; it is good for that organism to function well. Moreover, according to *Moral Prudentialism*, these interests generate moral protections. Thus, even bacteria, among the simplest forms of life, enjoy moral protections. If correct, this would mean that we have moral reasons to promote, protect, or not set back the interests of bacteria. Most would find this implication unacceptable. It is certainly wildly out of line with common sense morality, according to which we have no moral reason to refrain from killing bacteria. Indeed, this is the central problem with biocentric accounts of moral status. We remain open to the possibility that the *functional status* of bacteria and other very simple life forms may be determined by *Evolutionary Teleology* or some variant of it. But if it is, then either *Prudential Functionalism* or *Moral Prudentialism* will need to be rejected in order to avoid the implausible implication that simple organisms enjoy moral protections.

To avoid this implication, the proponent of the argument from evolutionary teleology might seek to restrict the scope of the argument so that it applies only to ‘higher organisms’, by which we mean organisms that can plausibly be taken to enjoy moral protections, for example, those that are conscious.^11^ There are three main ways in which this could be done. First, one could restrict the scope of *Evolutionary Teleology* so that it applies not to all evolved organisms, but only to evolved higher organisms. This might allow one to maintain that lower organisms possess no functional status. This is not a theoretically viable alternative, however, since it would result in a fragmented account of organismic function, drawing lines in unacceptably arbitrary ways from the standpoint of non-normative biological science. Second, we could restrict the scope of *Prudential Functionalism* so that it applies only to higher life forms—a move which might allow one to maintain that, even if lower organisms have a functional status, they have no interests. Third, one could restrict the scope of *Moral Prudentialism*. This would allow one to accept that lower organisms have functions and hence interests, but to deny that these interests generate any significant moral protections.

This is not the place to defend a view as to which of the latter two amendments should be made, but it should be noted that either amendment would constitute a significant concession. The new, restricted variant of the argument from evolutionary teleology would now no longer establish that the creation of simple artificial organisms, such as the JCVI bacterium, is morally significant. It would at most succeed in establishing the moral significance of creating *higher* artificial organisms, for only then would the problem of uncertain moral protections arise. Nevertheless, this is in itself an interesting conclusion. In addition, although on this argument the creation of simple artificial organisms would not count as morally significant, it might nevertheless be morally interesting in an indirect way: it might be an ominous sign of things to come, namely, the creation of higher artificial organisms.

But would a suitably restricted variant of the argument from teleology succeed even in demonstrating the moral significance of creating *higher* artificial organisms? There are two reasons to believe that it would not.

First, even in its restricted form, the argument has implausible implications. Since it assigns significant moral protections only to higher beings, it avoids the problem of scope—of implying that the range of organisms covered by moral protections is too wide. However, it has implausible implications for the content of those moral protections that it does ascribe.

Take the case of humans. According to the restricted argument from evolutionary teleology, humans enjoy moral protections that derive ultimately from the process via which they evolved. They are functioning well to the extent that they exhibit those traits that were favoured by natural selection in the evolution of their human ancestors. They thus have an interest in possessing those traits, and others have moral reasons to promote, protect or not set back the development and maintenance of those traits. But it is plausible that, for example, the evolutionarily given functions of the human brain include the generation of xenophobic psychological dispositions. These dispositions, and resulting hostility to out-groups, are characteristic of humans in a wide range of cultural environments and were probably favoured in our evolutionary past during periods of intense ecological competition between human groups. The restricted argument from evolutionary teleology thus implies that there are moral reasons to protect, promote or refrain from setting back the development and maintenance of xenophobic tendencies. This, we take it, is implausible.

The restricted variant of the argument from evolutionary teleology also has implausible implications for the content of the *interests* that it ascribes to higher beings. For example, it implies that a brain dead individual has an interest in having his body sustained by life support, even where he has previously rejected such support. Maintaining the body of a brain dead individual preserves some of his functioning—where functions are determined by *Evolutionary Teleology*—but most would find it difficult to accept that a brain dead individual has *any* interest in continued existence except perhaps where the individual previously wished to be maintained in such a state.

Not only does the argument from evolutionary teleology imply that organisms possess interests and enjoy moral protections that, intuitively, they do not possess and enjoy, it also makes these interests and protections implausibly contingent on facts about the evolutionary etiology of an organism. Consider the ‘swamp person’, who is exactly like an ordinary person in her non-genealogical properties but who, having arisen by chance from the primordial soup, has no history of selection to confer etiological biological functions. It seems very doubtful that, on discovering that someone whom we thought was an ordinary person was in fact a swamp person, we would change our views as to the interests or moral protections that the person possesses. Likewise, if synthetic biologists did manage to construct a human embryo entirely from scratch, and this developed into a human person, that person would, intuitively, be entitled to the same rights and privileges as another person, despite her curious origin. What matters, again, is not origin, but mental capacity.

The restricted argument from evolutionary teleology has counter-intuitive implications, both regarding *what* moral protections higher organisms enjoy and regarding *what determines* the existence of such protections.

In addition to these implausible implications, there is a further reason to doubt whether the argument from teleology, in either its general or restricted form, establishes the moral significance of creating artificial organisms. As with concerns about playing God, it is not clear that that the argument is sufficiently specific to *artificial* life.

According to both the general and restricted versions of the argument from evolutionary teleology, the problem with creating (higher) artificial organisms is that, because it would be unclear what moral protections they enjoy, there is a risk that they would be treated wrongfully. This problem arises, according to the argument, because artificial organisms are not evolved. As a result, their functional status is not determined by *Evolutionary Teleology*, and it is unclear what else determines their functional status. However, organisms derived from existing ones, such as via genetic modification or more radically by chimerization, are also not straightforwardly the products of natural selection.^12^ The final steps in the creation of genetically modified and chimeric entities were alterations brought about intentionally by rational agents. Thus, they too might seem to have an uncertain functional status.

On the one hand, the functional status of derived organisms could be determined by the same considerations that determine the functional status of non-living artefacts: that is, by whether they fulfil the purposes of the rational agents who designed and use them (although this might have unattractive implications of the sort suggested above). On the other hand, one might think that the functional status of derived organisms would be determined, at least in part, by considerations regarding their evolutionary past. For example, it could be that such an organism is functioning well just in case it possesses those traits which were favoured by natural selection in the evolution of its most proximate fully evolved ancestor. Alternatively, it could be that the functional status of derived organisms is determined by a mixture of evolutionary considerations and facts about the purposes of the agents who create and use them.

A reasonable lesson to draw from this discussion would be that, insofar as *Evolutionary Teleology* determines the functional status of natural, evolved organisms, the functional status of organisms derived through traditional genetic engineering practices is, like that of organisms created artificially, uncertain. If this is right, then concerns about creating beings with uncertain functional status are not specific to the creation of artificial organisms. Indeed they probably apply equally powerfully to the production all organisms (or all higher organisms) that are not wholly the product of undirected evolution and whose functional status therefore cannot straightforwardly be determined via *Evolutionary Teleology*.

We argued above that, insofar as the functional status of natural, evolved organisms is determined by *Evolutionary Teleology*, there is reason to deny that functional status determines moral status, for it is implausible that the moral protections enjoyed by such organisms are determined by their evolutionary genealogy. What matters for moral status are an organism’s non-genealogical properties, such as its mental capacities. We have now argued further that, even if evolutionary genealogy were relevant to moral status, the resulting uncertainty about the moral status of non-evolved creatures would apply as much to derived organisms as to artificial ones.

## 5 Conclusion

We have developed and evaluated three attempts to establish that the creation of artificial life is morally significant. These appealed respectively to concerns about playing God; to the possibility that the creation of such beings encourages reductionism and thus undermines the special status accorded to life; and to the thought that, since artificial life forms would have an uncertain functional status, they would also have an uncertain moral status.

We argued that none of these attempts succeeds in establishing the moral significance of creating artificial life. It does not, of course, follow that the creation of artificial life is morally insignificant, for there may be some other way of establishing its significance that we have missed. However, we believe that the attempts we have considered constitute the most charitable ways of understanding the most prominent arguments for the moral significance of artificial life. We thus believe that we have left the view that the creation of artificial life is morally significant on significantly shakier ground.

Though we have not argued for this view here, we believe that the capacity to create kinds of life that could never naturally exist does raise deep moral issues about the interests of those beings, their moral status, and the risks they pose to other beings. However, in our view, what matters, in answering those questions, is not how life is created but what non-genealogical properties it has.

## Footnotes

1 We use the term ‘organism’ as a term of art here. In common usage, there may be living entities that do not qualify as organisms (such as rainforests) and perhaps also organisms that are not living (such as deceased animals).

2 The term ‘natural’ is to be read as meaning ‘not supernatural’. This qualification is necessary because without it all organisms would be deemed artificial according to the standard creationist or intelligent design-theoretic view of life, insofar as God is presumed to be a rational agent—a result that is at odds with common usage of the term ‘artificial’. Note also that, as is clear from the definition, we do not take ‘artificial’ to mean inorganic or non-biological, although some might use the term in this way.

3 Note that it would not follow from the existence of moral reasons not to create artificial life that there is a decisive objection against creating it, since these reasons may be outweighed by moral reasons that speak in favour of creating artificial life.

4 We assume, for simplicity, that the process of creating an artificial organism is no more or less risky than the process of deriving a similar organism. Thus, any difference in risk will depend on the nature of the organism that is produced.

5 Similar considerations led the Task Group on Issues in Sample Return, a body of the Space Studies Board of the US National Research Council, to conclude in 1997 that the risk posed by the introduction of extra-terrestrial microbes to earth would be minimal.

6 For classic statements of a non-reductive physicalist position, see Davidson (1970) and Kim (1993).

7 We henceforth frame the concern as one about the actual ontological status of artificial organisms, but the argument could straightforwardly be reformulated in terms of their perceived ontological status.

8 We make no claim as to whether the arguments we make in this section apply to non-evolutionary accounts of biological function, such as dispositional, causal role or organizational maintenance approaches.

9 We thank Simon Rippon for pressing us to consider this puzzle.

10 It might be disputed whether a complex, highly cognitive creature that developed in the normal way from an artificial embryo would really count as artificial, since its development would depend heavily on input from other living systems (such as a social learning environment). Readers unconvinced that it would may instead imagine (more fantastically) a chimpanzee-like creature directly created from chemically simple, non-living components according to the design of a rational agent.

11 There would, of course, be significant disagreement about which beings fall into this category.

12 Chimeric organisms contain a mixture of cells from two or more species. They can be created by fusing the embryos of two different species.
